# Facility-Level Associations Between Use of a Digital Health Platform for Voluntary Counseling and Testing and HIV Testing Outcomes in the Urban Primary Health Care Centers of Guangzhou, China: Cross-Sectional Study

**DOI:** 10.2196/83662

**Published:** 2026-04-17

**Authors:** Ye Chen, Yu-Fei Wang, Yu-Zhou Gu, Xiao-Ru Fan, Yong-Heng Lu, Ju-Shuang Li, Jun Wang, Zhi-Ye Lin, Chun-Li Zhang, Meng-Die Li, Ning-Jun Ren, Zhi-Zhong Luo, Rui-Fei Zuo, Fu-Chuan Xiong, Yu-Tong Kang, Xiu-Rong Lin, Chun Hao

**Affiliations:** 1 Department of Medical Statistics School of Public Health Sun-Yat-Sen University Guangzhou China; 2 Sun Yat-Sen Global Health Institute Institute of State Governance| Institute of International and Regional Studies Sun-Yat-Sen University Guangzhou China; 3 Guangzhou Center for Disease Control and Prevention (Guangzhou Health Supervision Institute) Guangzhou China; 4 Liwan District Center for Disease Control and Prevention Guangzhou China; 5 Guangzhou Lingnan Community Support Center Guangzhou China; 6 Kangyuan Community Support Center of Yuexiu District Guangzhou China; 7 Department of Epidemiology and Health Statistics School of Public Health Hengyang Medical School Hengyang China; 8 Hospital of Stomatology Sun Yat-Sen University Guangzhou China; 9 Akeso Biopharma Co, Ltd Zhongshan China; 10 Department of Medical Record Management the Seventh Affiliated Hospital Sun Yat-Sen University Shenzhen China

**Keywords:** HIV testing, factor analysis, voluntary counseling and testing, health service, digital health

## Abstract

**Background:**

WellTest, a digital health platform designed to facilitate HIV voluntary counseling and testing (VCT) services, has been widely implemented in Guangzhou, China. However, the extent of its use by primary health care centers (PHCs) and the facility-level associations between WellTest use and HIV testing outcomes remain unclear.

**Objective:**

This study aimed to assess the use of WellTest at the facility level across PHCs in urban Guangzhou, China, and to explore the associations between WellTest use and HIV testing outcomes.

**Methods:**

We obtained data on VCT services visits from the National HIV/AIDS Prevention and Control Information System and the WellTest platform between January 1 and December 31, 2022. Two facility-level HIV testing outcomes, the HIV testing volume and HIV positivity rate, were calculated for each PHC. A structured questionnaire collected data on the PHCs’ characteristics. Multilevel negative binomial regression and zero-inflated gamma models were used to examine associations between WellTest use and 2 HIV testing outcomes at the facility level.

**Results:**

A total of 81 PHCs across 5 urban districts in Guangzhou were included. WellTest was used for 7997 active consultations and 7969 HIV tests that resulted in 157 newly diagnosed HIV cases, for an overall positivity rate of 2.0%, during 2022. The median share of clients booking via WellTest was 71% (IQR 34%-98%), and 81% (66/81) of PHCs offered online slots on all service days without mandates. PHCs that actively confirmed appointments had significantly lower HIV testing volumes compared with those that took no actions after clients scheduled appointments (incidence rate ratio 0.75, 95% CI 0.58-0.97; *P*=.03) but exhibited higher HIV positivity rates (β=.39, 95% CI 0.02-0.76; *P*=.04). Additionally, PHCs where ≥50% of clients used WellTest to schedule appointments showed higher HIV positivity rates than those with lower uptake (β=1.11; 95% CI 0.82-1.40; *P*<.001).

**Conclusions:**

WellTest showed substantial use among VCT clients and providers in urban Guangzhou PHCs, with greater use of digital appointments and counselor follow-up linked to increased HIV positivity rates. Strategies to optimize institutional adoption may help address stigma-related barriers, strengthen engagement in HIV testing, and support HIV case finding in primary health care settings.

## Introduction

HIV/AIDS has been a major public health problem since it was first reported in 1981. According to the Joint United Nations Program on HIV/AIDS [1], by the end of 2022, 39 million people were living with HIV. In China, by the end of 2022, there were an estimated 1.223 million people living with HIV, with 107,000 new infections and 30,000 AIDS-related deaths recorded in the same year [2]. At the provincial level, Guangdong Province has been one of the regions most affected by HIV/AIDS in China, accounting for 18.4% (11,806/64,170) of HIV infections in China [3,4] in 2018. Moreover, with many unaware of their HIV status, access to treatment was delayed and the risk of transmission increased [1]. As of 2024, 33.8% of the high-risk population in China remain unaware of their HIV status [5]. The UNAIDS 95-95-95 targets aim to eliminate the AIDS epidemic by 2030 [6], with the first target ensuring 95% of individuals living with HIV are aware of their status.

Voluntary counseling and testing (VCT) has been shown to contribute to the identification of potential individuals with HIV [7,8]. Since the establishment of a national VCT network in China in 2004, the number of VCT sites has grown to 11,319 [9]. However, workforce shortages and uneven distribution have impeded access to services [10]. To enhance accessibility, a 2009 national strategy promoted decentralizing VCT delivery to primary health care centers (PHCs) [11]. Despite this, the use of VCT services in PHCs remained low, hindered by insufficient awareness, stigma, and concerns over the quality of care [10,12-14].

Digital health interventions have shown promise in HIV/AIDS prevention due to their feasibility and acceptability. Tools such as social networks, online courses, and digital health apps have been proven to promote HIV testing [15-18], particularly among the high-risk population, by offering privacy, convenience, and efficiency [19]. Additionally, these interventions have improved the accessibility of disease testing [20,21]. However, research findings on whether digital health use can facilitate disease testing or screening at the facility level remains scarce [22].

Guangzhou, the capital of Guangdong Province and a major national central city with >18 million residents [23], has leveraged its advanced technological infrastructure to pioneer digital innovations aimed at enhancing access to VCT services within primary health care settings. In 2016, the Guangzhou Centers for Disease Control and Prevention (CDC) and Lingnan Partners Community Support Center (a community-based organization) developed WellTest, originating from the Lingnan website [24]. It is a digital platform for VCT services delivery for PHCs, by providing functions for online VCT services appointment booking, online-to-offline referral, clinic-based testing registration, and delivery of HIV test results. Since its expansion in 2018, the platform has enhanced VCT accessibility, facilitating 59,846 online consultations by 2023 and covering 83.6% of total VCT services visits in Guangzhou [25].

However, the implementation of a VCT digital health platform in PHCs and their impact on HIV testing outcomes at the facility level remain unclear. This study aims to examine the use of the WellTest platform in urban Guangzhou PHCs and its association with HIV testing outcomes at the facility level, providing novel evidence on how digital health use can optimize HIV testing services in primary health care settings.

## Methods

### Recruitment of Study Sites and Participants

The recruitment process was facilitated through the Guangzhou CDC. We identified all 81 public PHCs providing VCT services across 5 urban districts of Guangzhou (Tianhe, Yuexiu, Liwan, Baiyun, and Haizhu districts) in 2022. For each PHC, 1 primary VCT counselor was invited to complete the questionnaire survey for the data collection. Eligible counselors were those who agreed to participate in our study and had been working in VCT services for at least 6 months during the study period to ensure they had sufficient experience with the WellTest platform.

All identified PHCs and eligible counselors were formally invited to participate in this study with the help of Guangzhou CDC.

### Data Sources and Data Collection

Data on VCT services visits to these PHCs were obtained from two sources: the National HIV/AIDS Prevention and Control Information System and the digital health platform WellTest, spanning from January 1 to December 31, 2022. Individuals with incomplete HIV testing records were excluded from the analysis.

A questionnaire survey was conducted with VCT counselors from 81 PHCs to collect information on PHC characteristics, including digital health use, institutional details, and counselor profiles.

**Figure 1:**
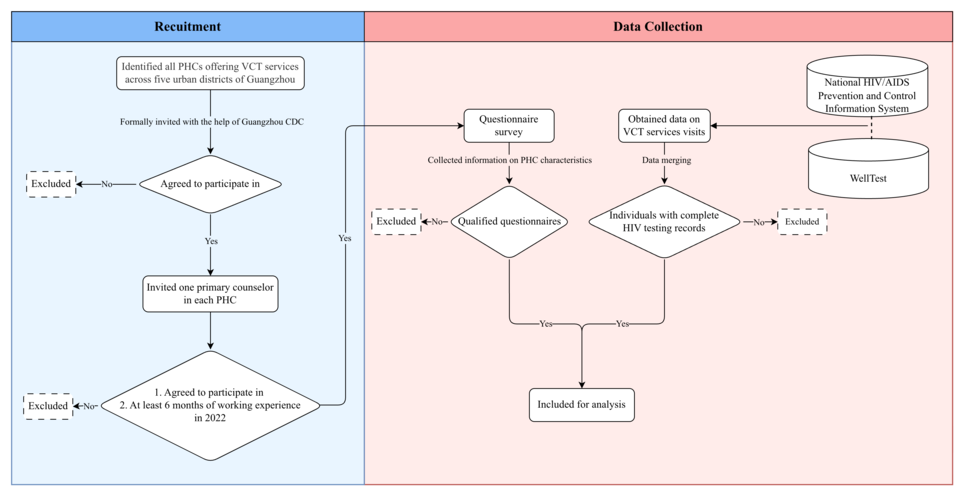
Flowchart of the recruitment and data collection process. CDC: Centers for Disease Control and Prevention; PHC: primary health care center; VCT: voluntary counseling and testing.

The recruitment and data collection flow is depicted in Figure 1.

### Measurements

Two facility-level HIV testing outcomes were assessed for each PHC:

- HIV testing volume; the total number of HIV tests conducted at each PHC
- HIV positivity rate; calculated as the number of newly diagnosed HIV cases/the number of HIV tests conducted

We merged the two aforementioned databases and then calculated the number of HIV tests conducted and the number of newly diagnosed HIV cases for each PHC providing VCT services in 2022.

Associated factors include digital health use, institutional details, and counselor profiles. Digital health use included the proportion of clients scheduling VCT appointments through WellTest, whether to set online appointment slots for all VCT service days via WellTest, and counselor responses to WellTest appointments (take no actions or confirm appointment by contacting clients via phone or SMS text message). Counselor profiles encompassed age, gender, educational level, major, working experience, and proportion of VCT services within their workload. PHCs’ institutional details consisted of five domains. The first is organizational structure, that is, the ownership type of the PHC (public or private) and whether the PHC prioritizes VCT services in routine practice. The second is human resources, that is, the number of VCT counselors and the frequency of counselor turnover during the study period. The third is infrastructure, that is, the availability of dedicated phlebotomy rooms and the availability of private and dedicated counseling spaces. The fourth is location, that is, the walking time to the nearest subway station (≤15 minutes or >15 minutes, based on the commonly used standard of approximately 15 minutes per km, which is generally considered a walkable distance). The distance to the nearest subway station and geographical coordinates were obtained using Amap, a widely used digital mapping service in China. The fifth is service delivery, that is, the average turnaround time for HIV test results, whether services were available on weekends, the number of times counselors were unable to provide VCT services for personal reasons in 2022, and the availability of posttest gifts (HIV prevention kits, including condoms, lubricants, and informational materials) for testers.

### Statistical Analysis

Two maps were created to illustrate the HIV testing volume and HIV testing rate for each PHC. Descriptive statistics were used to depict VCT digital health use and the characteristics of counselors and PHCs. Given that HIV testing volume at the facility level exhibited overdispersion and the HIV positivity rate was highly right skewed with an excess of zeros, we applied multilevel negative binomial regression [26] and zero-inflated gamma models [27]. These methods were used to examine the associations between WellTest use and HIV testing outcomes, including testing volume and positivity rate, across primary health care facilities. The univariate analysis was conducted to assess associations between VCT digital health use, the characteristics of VCT counselors, and PHCs with 2 HIV testing outcomes. Subsequently, variables at a significance level of *P*<.10 from the univariate analysis, along with 3 variables of the “digital health use” domain, were included in the multivariable analysis to investigate the adjusted association between the use of WellTest and HIV testing among PHCs in urban Guangzhou. The population under the jurisdiction of each PHC, representing the facility-level service catchment area and underlying demand of HIV testing, was included as a confounder. *P*<.05 was considered statistically significant in the multivariable analysis. To test the robustness of our findings, we conducted a sensitivity analysis excluding data from Liwan district due to its limited sample size (n=2), following the same steps as the primary analysis. All analyses were performed using R (version 4.4.3; R Foundation for Statistical Computing).

### Ethical Considerations

This study was approved by the ethics committee of Sun Yat-sen University (Institutional Review Board number 054/19; February 28, 2019). All questionnaires were completed with the counselors’ informed consent, and the analysis of data on VCT services visits was approved by Guangzhou CDC, with no additional ethical review required. All data used were deidentified and analyzed solely within the research team to ensure the confidentiality and privacy of the data.

## Results

### Recruitment

All PHCs offering VCT services in the urban area of Guangzhou in 2022 were included in our study (N=81), and all 81 invited counselors from the respective PHCs agreed to participate in this study. No PHCs or counselors were excluded from the analysis due to poor questionnaire quality or logical inconsistencies.

### HIV Testing Among PHCs Offering VCT Services

**Figure 2:**
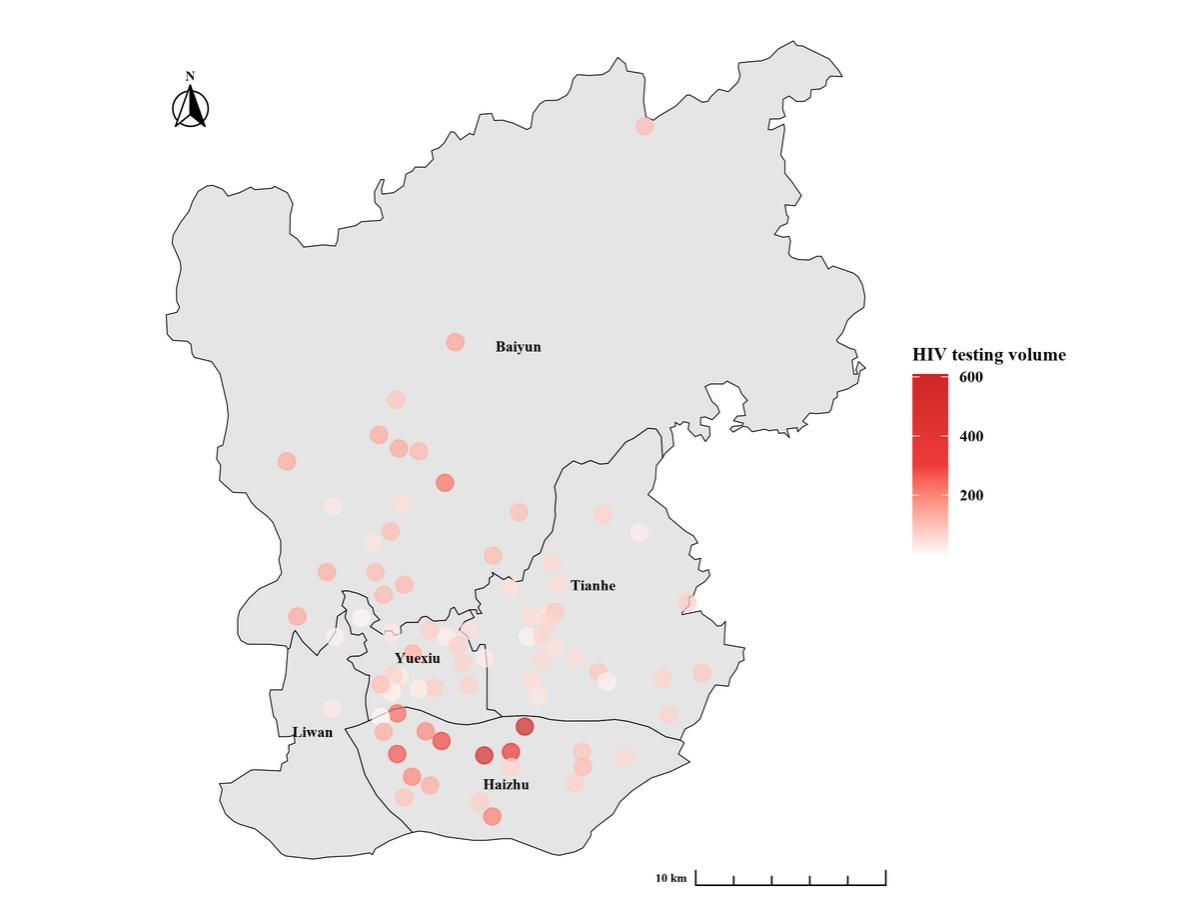
HIV testing volumes for each primary health care center offering voluntary counseling and testing services in the urban area of Guangzhou, China, in 2022.

**Figure 3:**
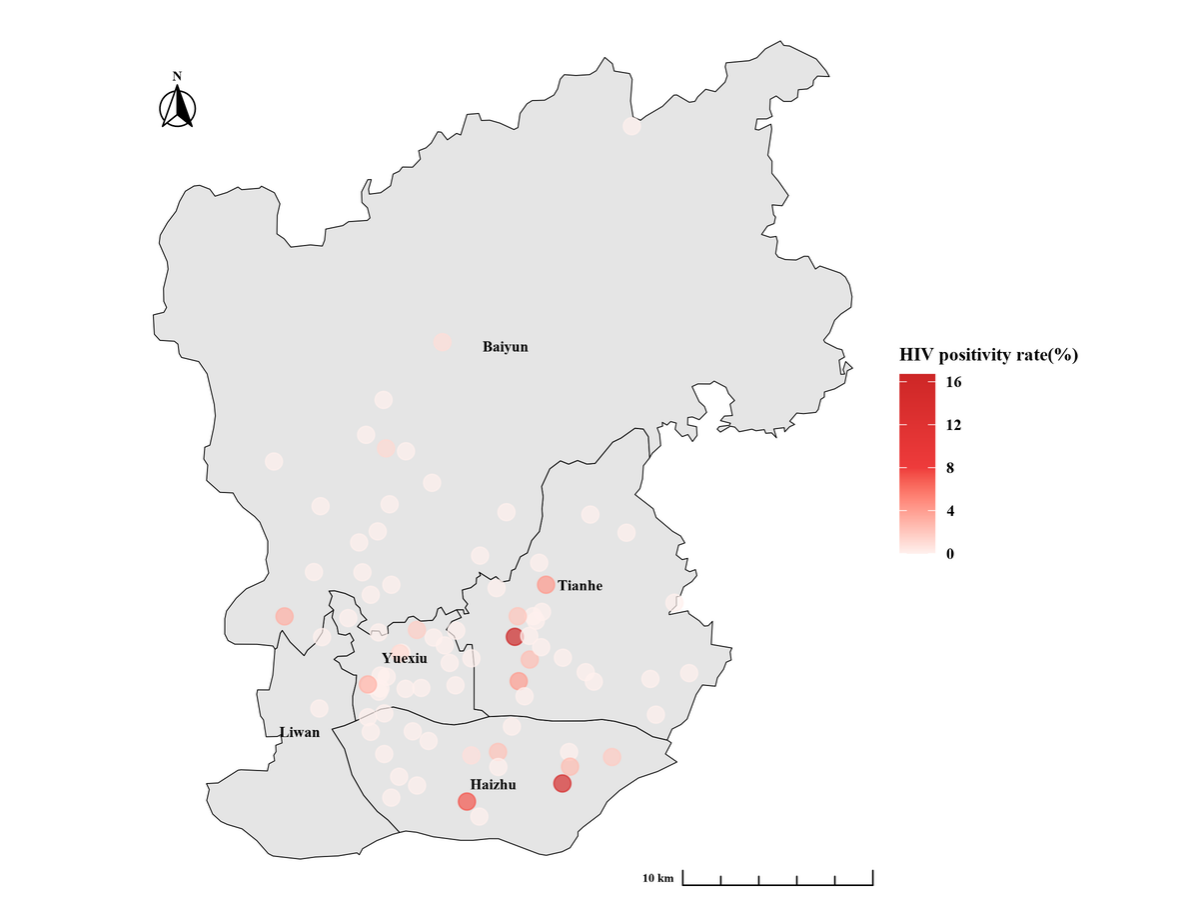
HIV positivity rates for each primary health care center offering voluntary counseling and testing services in the urban area of Guangzhou, China, in 2022.

After excluding 5 individuals with incomplete HIV testing records, 81 urban PHCs of Guangzhou offered a total of 7997 person-times of consultations and 7969 HIV tests, identified 157 new diagnoses, and resulted in an HIV-positive rate of 2.0% in 2022. Figure 2 shows the HIV testing volumes for each urban PHC in 2022, varying from 2 to 609 (median 71, IQR 45-112). Figure 3 presents the HIV-positive rates for each urban PHC in 2022, ranging from 0% to 16.7% (median 0, IQR 0-0).

### Digital Health Use for VCT Services Delivery

Across the 81 PHCs, the median number of clients actively seeking VCT services was 71 (IQR 44-112). Among them, a median of 30 (IQR 10-62) clients scheduled appointments via WellTest, corresponding to a high proportion of scheduling VCT appointments through WellTest (median 71%, IQR 34%-98%). On the provider side, the median number of days per week offering VCT services was 3 (IQR 2-5) days, and a median of 3 (IQR 2-5) appointment days weekly was set in WellTest. The median proportion of service days with available online slots was 100% (IQR 100%-100%).

As shown in Table 1, most PHCs (66/81, 81%) opened online appointment slots on all service days. After online appointments were made, nearly half (39/81, 48%) reported counselor follow-up.

**Table 1:** Characteristics of primary health care centers offering voluntary counseling and testing services in the urban area of Guangzhou, China, in 2022 (N=81).

Characteristics					Primary health care centers
**Digital health use**					
	**Proportion of clients scheduling VCT** ^a^ **services through WellTest (%), median (IQR)**				71 (34-98)
	**Set online appointment slots on all VCT service days via WellTest, n (%)**				
		No		15 (19)	
		Yes		66 (81)	
	**Action taken by counselors upon receiving appointments from WellTest, n (%)**				
		Take no action		42 (52)	
		Confirm appointment (contacting client via phone or text message)		39 (48)	
**Institutional details**					
	**Organizational structure**				
		**Ownership, n (%)**			
			Public ownership	57 (70)	
			Private ownership	24 (30)	
		**Institutional prioritization of VCT services in routine practice, n (%)**			
			No	27 (33)	
			Yes	54 (67)	
	**Human resources**				
		**Number of counselors, n (%)**			
			1	35 (43)	
			2 or 3	46 (57)	
		**Counselor turnover in the past year, n (%)**			
			No	62 (77)	
			Yes	19 (23)	
	**Infrastructure**				
		**Dedicated phlebotomy rooms, n (%)**			
			No	42 (52)	
			Yes	39 (48)	
		**Dedicated and private counseling rooms, n (%)**			
			No	29 (36)	
			Yes	52 (64)	
	**Location**				
		**Walking time to the nearest subway station (minutes), n (%)**			
			≤15	43 (53)	
			>15	38 (47)	
		**Administrative district, n (%)**			
			Tianhe district	23 (28)	
			Haizhu district	19 (24)	
			Baiyun district	19 (24)	
			Liwan district	2 (2)	
			Yuexiu district	18 (22)	
	**Service delivery**				
		**Turnaround time of HIV test results, n (%)**			
			Within 1 day	65 (80)	
			More than 1 day	16 (20)	
		**Availability of VCT services on weekends, n (%)**			
			No	57 (70)	
			Yes	24 (30)	
		**Number of times counselors were unable to provide VCT services for personal reasons last year, n (%)**			
			0	38 (47)	
			<5	32 (39)	
			≥5	11 (14)	
		**Posttest gifts for testers, n (%)**			
			No	3 (4)	
			Yes	78 (96)	
**Counselor profiles**					
	**Age (years), n (%)**				
		≤35		46 (57)	
		>35		35 (43)	
	**Gender, n (%)**				
		Male		30 (37)	
		Female		51 (63)	
	**Educational level: bachelor’s degree or higher, n (%)**				
		No		17 (21)	
		Yes		64 (79)	
	**Major in public health, n (%)**				
		No		40 (49)	
		Yes		41 (51)	
	**Work experience (years), n (%)**				
		≤3		40 (49)	
		>3		41 (51)	
	**Proportion of VCT services within the counselor’s workload, n (%)**				
		≤50%		73 (90)	
		>50%		8 (10)	

^a^VCT: voluntary counseling and testing.

### Institutional Details and Counselor Profiles of PHCs Offering VCT Services

Table 1 summarizes institutional details and counselor profiles of PHCs. The distribution of PHCs was relatively balanced: Tianhe had the largest share (23/81, 28%), followed by Haizhu (19/81, 24%), Baiyun (19/81, 24%), Yuexiu (18/81, 22%), and Liwan, the smallest (2/81, 2%). Most PHCs were publicly owned (57/81, 70%) and prioritized VCT services in their routine practice (54/81, 67%). Nearly half had only 1 counselor (35/81, 43%), while staff turnover was uncommon (19/81, 23%). About half lacked a dedicated phlebotomy room (42/81, 52%), but most had a private counseling room (52/81, 64%). Over half were within 15 minutes of the nearest subway station (43/81, 53%). Most could return HIV results within a day (65/81, 80%) but did not provide weekend VCT services (57/81, 70%). About half (38/81, 47%) of the PHCs reported no inability to offer VCT services by counselors due to personal reasons last year, and almost all provided posttest gifts for testers (78/81, 96%).

Among 81 VCT counselors, most were female (51/81, 63%) and aged ≤35 years (46/81, 57%). The majority held a bachelor’s degree or higher (64/81, 79%), about half had a public health background (41/81, 51%) and >3 years’ experience (41/81, 51%), and most spent >50% of their workload on VCT services (73/81, 90%).

### Facility-Level Associations Between WellTest Use and HIV Testing Volume

The null model for the HIV testing volume indicated a statistically significant random effect at the cluster level (*P*<.001), suggesting that HIV testing volumes were nested within administrative districts, showing significant interdistrict variation. In the univariate analysis, 3 variables (number of times counselors were unable to provide VCT services for personal reasons last year, counselors’ age, and proportion of VCT services within the counselor’s workload) were significantly associated with facility-level HIV testing volume (*P*<.10). Results of the sensitivity analysis are consistent with those of the primary analysis. Further details of the primary analysis and sensitivity analysis are presented in Tables S1 and S3 in Multimedia Appendix 1, respectively.

The results of the multivariate analysis for HIV testing volume are presented in Table 2. On the basis of a multilevel negative binomial regression model, PHCs that confirmed appointments had significantly lower HIV testing volumes compared with those that took no actions after clients made appointments (incidence rate ratio 0.75, 95% CI 0.58-0.97; *P*=.03), indicating a 25% reduction in HIV testing volume associated with appointment confirmation. Table 2 also illustrates results of the sensitivity analysis, which are consistent with the primary analysis.

**Table 2:** Unadjusted and multivariable analysis^a^ of the facility-level associations of the use of WellTest with HIV testing volume among primary health care centers (PHCs) offering voluntary counseling and testing services in the urban area of Guangzhou, China, in 2022.

Variables				Facility-level HIV testing volume, median (IQR)		IRR^b^ (95% CI)		*P* value		IRR_m_^c^ (95% CI)		*P* value
**Primary analysis**												
	**Proportion of clients scheduling VCT^d^ services through WellTest**											
		<50%	106 (70-142)		Reference		—^e^		Reference		—	
		≥50%	58 (34-87)		0.83 (0.60-1.15)		.27		0.93 (0.69-1.27)		.66	
	**Set online appointment slots on all VCT service days via WellTest**											
		No	83 (63-121)		Reference		—		Reference		—	
		Yes	70 (38-110)		1.00 (0.68-1.46)		.98		1.09 (0.80-1.50)		.58	
	**Actions taken by counselors upon receiving appointments from WellTest**											
		Take no actions	79 (48-138)		Reference		—		Reference		—	
		Confirm appointments (contacting clients via phone or SMS text message)	69 (40-96)		0.69 (0.52-0.92)		.01		0.75 (0.58-0.97)		.03	
**Sensitivity analysis**												
	**Proportion of clients scheduling VCT services through WellTest**											
		<50%	106 (70-142)		Reference		—		Reference		—	
		≥50%	61 (35-88)		0.84 (0.61-1.17)		.30		0.94 (0.69-1.28)		.70	
	**Set online appointment slots on all VCT service days via WellTest**											
		No	83 (63-121)		Reference		—		Reference		—	
		Yes	70 (46-112)		1.02 (0.70-1.49)		.93		1.11 (0.81-1.52)		.53	
	**Actions taken by counselors upon receiving appointments from WellTest**											
		Take no actions	83 (48-139)		Reference		—		Reference		—	
		Confirm appointments (contacting clients via phone or SMS text message)	70 (46-97)		0.69 (0.52-0.92)		.01		0.75 (0.58-0.98)		.03	

^a^All models were adjusted by the population under the jurisdiction of each primary health care enter.

^b^IRR: unadjusted incidence rate ratio; IRR=e^β^.

^c^IRR_m_: multivariate-adjusted incidence rate ratio.

^d^VCT: voluntary counseling and testing.

^e^Not applicable.

### Facility-Level Associations Between WellTest Use and HIV Positivity Rate

For HIV positivity rate, the null model revealed that the random effect at the cluster level was not statistically significant (*P*>.99), indicating no district-level clustering. In the univariate analysis, 5 variables (the availability of dedicated phlebotomy rooms, the availability of private and dedicated counseling spaces, administrative district, availability of posttest gifts for testers, and proportion of VCT services within the counselor’s workload) were significantly associated with facility-level HIV positivity rate at the significance level of *P*<.10. The sensitivity analysis produced results consistent with the primary analysis. Detailed findings of the primary analysis and sensitivity analysis are presented in Tables S2 and S4 in Multimedia Appendix 1, respectively.

Table 3 presents the results of multivariate analysis for the HIV positivity rate. According to the zero-inflated gamma distribution model adjusted by relative factors, PHCs in which ≥50% of clients scheduled VCT services through WellTest demonstrated higher positivity rates than those with <50% who scheduled through the platform (β=.39, 95% CI 0.02-0.76; *P*=.04). Additionally, PHCs that contacted clients via phone or SMS text message to confirm appointments showed a significantly higher HIV positivity rate compared with those that took no action (β=1.11, 95% CI 0.82-1.40; *P*<.001). Table 3 also presents results of the sensitivity analysis, which are consistent with the primary analysis.

**Table 3:** Unadjusted and multivariable analysis^a^ of the facility-level associations of the use of WellTest with the HIV positivity rate among primary health care centers offering voluntary counseling and testing services in the urban area of Guangzhou, China, 2022.

Variables				Facility-level HIV positivity, median (IQR), %		β^b^ (95% CI)		*P* value		β_m_^c^ (95% CI)		*P* value
**Primary analysis**												
	**Proportion of clients scheduling VCT** ^d^ **services through WellTest**											
		<50%	0.0 (0.0-0.0)		Reference		—^e^		Reference		—	
		≥50%	0.0 (0.0-0.0)		0.07 (−0.78 to 0.93)		.87		0.39 (0.02 to 0.76)		.04	
	**Set online appointment slots on all VCT service days via WellTest**											
		No	0.0 (0.0-0.0)		Reference		—		Reference		—	
		Yes	0.0 (0.0-0.0)		−0.42 (−1.48 to 0.63)		.44		−0.42 (−0.85 to 0.01)		.06	
	**Actions taken by counselors upon receiving appointments from WellTest**											
		Take no actions	0.0 (0.0-0.0)		Reference		—		Reference		—	
		Confirm appointments (contacting clients via phone or SMS text message)	0.0 (0.0-0.0)		1.07 (0.39 to 1.76)		.003		1.11 (0.82 to 1.40)		<.001	
**Sensitivity analysis**												
	**Proportion of clients scheduling VCT services through WellTest**											
		<50%	0.0 (0.0-0.0)		Reference		—		Reference		—	
		≥50%	0.0 (0.0-0.0)		0.07 (−0.78 to 0.93)		.87		0.39 (0.02 to 0.77)		.04	
	**Set online appointment slots on all VCT service days via WellTest**											
		No	0.0 (0.0-0.0)		Reference		—		Reference		—	
		Yes	0.0 (0.0-0.0)		−0.42 (−1.48 to 0.63)		.44		−0.42 (−0.84 to 0.00)		.055	
	**Actions taken by counselors upon receiving appointments from WellTest**											
		Take no actions	0.0 (0.0-0.0)		Reference		—		Reference		—	
		Confirm appointments (contacting clients via phone or SMS text message)	0.0 (0.0-0.0)		1.07 (0.39 to 1.76)		.003		1.11 (0.81 to 1.42)		<.001	

^a^All models were adjusted by the population under the jurisdiction of each primary health care center.

^b^β: unadjusted β.

^c^β_m_: multivariate-adjusted β.

^d^VCT: voluntary counseling and testing.

^e^Not applicable.

## Discussion

### Principal Findings

Among 81 PHCs offering VCT services in urban Guangzhou in this study, the median proportion of clients using WellTest for appointments was 71% (IQR 34%-98%), while 81% (66/81) of PHCs set online appointment slots on all service days without government mandates. For the client side, facilities with more digital bookings had higher HIV positivity rates. For the provider side, those with active counselors follow-up also had higher positivity rates but lower overall testing volume. These findings indicated strong demand for WellTest as an appointment platform from both providers and clients and suggested that proactive counselor engagement may improve the efficiency of HIV case detection (as reflected by higher positivity rates), even if total testing volume declines.

In the absence of top-down administrative arrangements, WellTest experienced spontaneous and sustained uptake among providers and clients [25]. To our best knowledge, this is the first facility-level report of VCT service digital platform adoption globally. Previous research has shown that digital platforms could effectively facilitate HIV testing at the individual level [15,28], while this study showed the same finding at the facility level. In addition, digital platforms have been found to promote health information dissemination and enhance HIV testing willingness [29-31]. As stigma is considered as a main psychosocial barrier to HIV testing [32], using digital appointment platforms at primary health care facilities might increase accessibility to HIV testing while mitigating stigma associated with walk-in visits. This indicated digital appointment platforms might facilitate access to stigma-sensitive health services.

In terms of appointment management, our findings suggested that the counselor-initiated engagement, such as confirming online appointments via phone calls or SMS text messages, might be associated with a higher HIV positivity rate at the facility level, indicating improved case-detection efficiency despite fewer total tests. Very few studies have reported that active follow-up by health care providers may inadvertently create barriers that influence clients’ willingness to undergo HIV testing. Psychosocial barriers such as stigma [32-35], anxiety, and fear of test results [36,37] as major barriers to HIV testing and limited social support [38] deter testing, while high-risk individuals are more motivated to enroll for testing [39]. Although WellTest may play a role in improving the VCT access, the action of confirming appointments may create psychological or logistical hurdles, increasing anxiety and dropouts. However, this step may also filter out less committed or low-risk clients, raising the positivity rate and improving testing efficiency at PHCs at the facility level. Clients seeking testing often experience anxiety, depression, and emotional distress [40]. Counseling and support, as essential components of VCT, have been shown to reduce such psychological burdens [41]and enhance the client experience [42]. As clients often experience anxiety and distress, integrating brief psychological support into the appointment confirmation stage might help enhance retention and engagement. Adding automated reminders in WellTest may also nudge clients without interpersonal pressure, reduce burden, and improve comfort, willingness, and attendance while preserving support and privacy, which is crucial in HIV testing.

Furthermore, the analysis revealed that a higher proportion of clients who scheduled VCT services through digital health platforms had higher HIV case–detection rates at the facility level. This suggested that WellTest may attract higher-risk individuals who were more motivated to seek testing. A prior study has shown that personalized outreach via social media might effectively promote testing among high-risk populations [43]. Embedding peer-to-peer features, for example, privacy-preserving “share with a friend or partner” prompts and referral links, within WellTest might boost uptake and further improve case findings.

### Limitations

This study has some limitations. As a cross-sectional study, it cannot establish causality, consistent with similar publications [44-46]. Furthermore, it spanned the entirety of the year 2022 when Guangzhou experienced intermittent COVID-19 pandemic restrictions, which may have affected HIV testing patterns, potentially limiting the generalizability of our findings to nonpandemic conditions. As this study was conducted in the urban area of Guangzhou, a leading metropolitan hub in China, the findings may not be directly applicable to rural or resource-limited regions. Additionally, several potential facility-level and individual-level confounding factors could not be fully addressed in this analysis due to data availability and models’ stability. Future studies using longitudinal data that link to more related confounders are needed to better clarify the causal relationship between digital platform use and HIV testing outcomes. Notably, while the WellTest platform effectively engages high-risk populations most in need of VCT services, digital health platforms may encounter digital disparities among older adult populations. Nonetheless, the data provide valuable evidence on facility-level PHC service delivery and digital platform use under real-world conditions and offer suggestions for improving VCT digital platforms.

### Conclusions

This study demonstrated the substantial use of WellTest among clients and providers in Guangzhou’s urban PHCs and identified its potential role in improving HIV testing services delivery in primary health care settings. To further optimize HIV testing services, the integration of psychological support into appointment confirmation, with automated reminders to reduce client burden and peer-referral features via social networks, could be considered. Such strategies may help address psychosocial barriers, strengthen engagement in HIV testing, and support case finding in primary health care settings.

## Data Availability

The datasets generated or analyzed during this study, obtained from Guangzhou Centers for Disease Control and Prevention, are not publicly available due to confidentiality and privacy but are available from the corresponding author on reasonable request.

## Appendix

Multimedia Appendix 1 Results of the primary and sensitivity analysis for associations between institutional details and counselor profiles and facility-level HIV testing outcomes among primary health care centers offering voluntary counseling and testing services in the urban area of Guangzhou, China, in 2022.

## Abbreviations

CDC: Centers for Disease Control and Prevention
PHC: primary health care center
VCT: voluntary counseling and testing
