# Supplementary material for: Facility-Level Associations Between Use of a Digital Health Platform for Voluntary Counseling and Testing and HIV Testing Outcomes in the Urban Primary Health Care Centers of Guangzhou, China: Cross-Sectional Study
Source: J Med Internet Res. 2026 Apr 17;28:e83662. doi: 10.2196/83662 (PMC13135155; doi:10.2196/83662)
Supplement: Multimedia Appendix 1 [file jmir_v28i1e83662_app1.docx]

Table S1. Primary analysis: Unadjusted and multivariable analysis^a^ of the facility-level associations of institutional details and counselor profiles with HIV testing volume among primary health care centers (PHCs) in the urban area of Guangzhou, China, 2022.

| Variables | | | | Facility-level HIV testing volume, Median (IQR) | IRR^b^ (95%CI) | *P* value | IRR_m_^c^ (95%CI) | *P* value |
| --- | --- | --- | --- | --- | --- | --- | --- | --- |
| **Institutional details** | | | | | | | | |
|  | **Organizational structure** | | | | | | | |
|  |  | **Ownership, n (%)** | |  |  |  |  |  |
|  |  |  | Public ownership | 68 (30-129) | Reference | —^e^ | — | — |
|  |  |  | Private ownership | 75 (65-105) | 0.88 (0.64-1.22) | .45 | — | — |
|  |  | **Institutional prioritization of VCT^d^ services in routine practice, n (%)** | | | | | | |
|  |  |  | No | 68 (35-89) | Reference | — | — | — |
|  |  |  | Yes | 81 (48-126) | 1.21 (0.90-1.65) | .22 | — | — |
|  | **Human resources** | | |  |  |  |  |  |
|  |  | **Number of counselors, n (%)** | | | | | | |
|  |  |  | 1 | 71 (42-107) | Reference | — | — | — |
|  |  |  | 2 or 3 | 74 (46-129) | 1.14 (0.85-1.52) | .37 | — | — |
|  |  | **Counselor turnover in the past year, n (%)** | | | | | | |
|  |  |  | No | 74 (47-114) | Reference | — | — | — |
|  |  |  | Yes | 61 (32-97) | 0.99 (0.70-1.42) | .98 | — | — |
|  | **Infrastructure** | | |  |  |  |  |  |
|  |  | **Dedicated phlebotomy rooms, n (%)** | | | | | | |
|  |  |  | No | 70 (48-110) | Reference | — | — | — |
|  |  |  | Yes | 76 (35-121) | 0.95 (0.72-1.27) | .75 | — | — |
|  |  | **Dedicated and private counseling rooms, n (%)** | | | | | | |
|  |  |  | No | 68 (34-83) | Reference | — | — | — |
|  |  |  | Yes | 86 (50-136) | 1.25 (0.90-1.72) | .17 | — | — |
|  | **Location** | | |  |  |  |  |  |
|  |  | **Walking time to the nearest subway station (minutes), n (%)** | | | | | | |
|  |  |  | ≤15 | 68 (32-107) | Reference | — | — | — |
|  |  |  | >15 | 86 (48-114) | 1.05 (0.76-1.43) | .78 | — | — |
|  |  | **Administrative district, n (%)** | |  |  |  |  |  |
|  |  |  | Tianhe district | 52 (44-68) | — | — | — | — |
|  |  |  | Haizhu district | 131 (84-253) | — | — | — | — |
|  |  |  | Baiyun district | 111 (102-136) | — | — | — | — |
|  |  |  | Liwan district | 22 (18-26) | — | — | — | — |
|  |  |  | Yuexiu district | 40 (22-71) | — | — | — | — |
|  | **Service delivery** | | |  |  |  |  |  |
|  |  | **Turnaround time for HIV test results, n (%)** | | | | | | |
|  |  |  | Within 1 day | 72 (45-112) | Reference | — | — | — |
|  |  |  | More than 1 day | 68 (44-99) | 0.99 (0.69-1.43) | .98 | — | — |
|  |  | **Availability of VCT services on weekends, n (%)** | | | | | | |
|  |  |  | No | 68 (30-104) | Reference | — | — | — |
|  |  |  | Yes | 102 (68-136) | 1.01 (0.70-1.45) | .95 | — | — |
|  |  | **Number of times counselors were unable to provide VCT services for personal reasons last year, n (%)** | | | | | | |
|  |  |  | 0 | 84 (57-125) | Reference | — | Reference | — |
|  |  |  | <5 | 66 (43-112) | 0.85 (0.63-1.15) | .29 | 0.84 (0.65-1.09) | .19 |
|  |  |  | ≥5 | 50 (22-105) | 0.66 (0.43-1.01) | .054 | 0.74 (0.50-1.10) | .14 |
|  |  | **Posttest gifts for testers, n (%)** | |  |  |  |  |  |
|  |  |  | No | 71 (64-171) | Reference | — | — | — |
|  |  |  | Yes | 72 (43-112) | 0.76 (0.36-1.60) | .46 | — | — |
| **Counselor profiles** | | | | | | | | |
|  | **Age (years), n (%)** | | |  |  |  |  |  |
|  |  | ≤35 | | 72 (31-106) | Reference | — | Reference | — |
|  |  | >35 | | 71 (52-114) | 1.36 (1.02-1.80) | .04 | 1.17 (0.90-1.52) | .25 |
|  | **Gender, n (%)** | | |  |  |  |  |  |
|  |  | Male | | 58 (31-130) | Reference | — | — | — |
|  |  | Female | | 75 (52-109) | 1.03 (0.76-1.39) | .86 | — | — |
|  | **Educational level：Bachelor’s degree or higher, n (%)** | | | | | | | |
|  |  | No | | 75 (48-99) | Reference | — | — | — |
|  |  | Yes | | 71 (40-129) | 1.00 (0.70-1.43) | .998 | — | — |
|  | **Major in Public Health, n (%)** | | | | | | | |
|  |  | No | | 76 (53-113) | 1.04 (0.77-1.39) | .82 | — | — |
|  |  | Yes | | 68 (34-111) | Reference | — | — | — |
|  | **Work experience (years), n (%)** | | | | | | | |
|  |  | ≤3 | | 68 (50-109) | Reference | — | — | — |
|  |  | >3 | | 72 (36-112) | 1.06 (0.79-1.43) | .69 | — | — |
|  | **Proportion of VCT services within the counselor's workload, n (%)** | | | | | | | |
|  |  | ≤50% | | 71 (47-115) | Reference | — | Reference | — |
|  |  | >50% | | 70 (23-100) | 0.65 (0.40-1.03) | .07 | 0.71 (0.45-1.12) | .14 |

^a^All models were adjusted by the population under the jurisdiction of each PHC.

^b^IRR: unadjusted incidence rate ratio, IRR =e^β^.

^c^IRR_m_: multivariate-adjusted incidence rate ratio.

^d^VCT: voluntary counseling and testing.

^e^Not applicable.

Table S2. Primary analysis: Unadjusted and multivariable Gamma analysis^a^ of the facility-level associations of institutional details and counselor profiles with HIV positivity rate among primary health care centers (PHCs) offering VCT services in the urban area of Guangzhou, China, 2022.

| Variables | | | | Facility-level HIV positivity rate, median (IQR), % | β^b^ (95%CI) | *P* value | β_m_^c^ (95%CI) | *P* value |
| --- | --- | --- | --- | --- | --- | --- | --- | --- |
| **Institutional details** | | | | | | | | |
|  | **Organizational structure** | | |  |  |  |  |  |
|  |  | **Ownership, n (%)** | | | | | | |
|  |  |  | Public ownership | 0.0 (0.0-0.0) | Reference | —^e^ | — | — |
|  |  |  | Private ownership | 0.0 (0.0-1.9) | 0.25 (–0.56 to 1.06) | .55 | — | — |
|  |  | **Institutional prioritization of VCT^d^ services in routine practice, n (%)** | | | | | | |
|  |  |  | No | 0.0 (0.0-0.0) | Reference | — | — | — |
|  |  |  | Yes | 0.0 (0.0-0.7) | –0.79 (–1.80 to 0.21) | .13 | — | — |
|  | **Human resources** | | |  |  |  |  |  |
|  |  | **Number of counselors, n (%)** | | | | | | |
|  |  |  | 1 | 0.0 (0.0-0.0) | Reference | — | — | — |
|  |  |  | 2 or 3 | 0.0 (0.0–0.0) | 0.12 (–0.70 to 0.94) | .77 | — | — |
|  |  | **Counselor turnover in the past year, n (%)** | | | | | | |
|  |  |  | No | 0.0 (0.0-0.0) | Reference | — | — | — |
|  |  |  | Yes | 0.0 (0.0-0.0) | –0.34 (–1.41 to 0.72) | .53 | — | — |
|  | **Infrastructure** | | |  |  |  |  |  |
|  |  | **Dedicated phlebotomy rooms, n (%)** | | | | | | |
|  |  |  | No | 0.0 (0.0-0.0) | Reference | — | Reference | — |
|  |  |  | Yes | 0.0 (0.0-0.0) | –0.76 (–1.51 to –0.01) | .051 | 0.85 (0.61 to 1.10) | <.001 |
|  |  | **Dedicated and private counseling rooms, n (%)** | | | | | | |
|  |  |  | No | 0.0 (0.0-0.0) | Reference | — | Reference | — |
|  |  |  | Yes | 0.0 (0.0-0.0) | –1.33 (–1.98 to –0.68) | <.001 | –1.47 (–1.75 to –1.19) | <.001 |
|  | **Location** | | |  |  |  |  |  |
|  |  | **Walking time to the nearest subway station (minutes), n (%)** | | | | | | |
|  |  |  | ≤15 | 0.0 (0.0-0.0) | Reference | — | — | — |
|  |  |  | >15 | 0.0 (0.0-0.0) | 0.07 (–0.75 to 0.89) | .86 | — | — |
|  |  | **Administrative district, n (%)** | | | | | | |
|  |  |  | Tianhe district | 0.0 (0.0-0.0) | Reference | — | Reference | — |
|  |  |  | Haizhu district | 0.0 (0.0-1.3) | –0.21 (–1.08 to 0.67) | .65 | –0.53 (–0.79 to –0.28) | <.001 |
|  |  |  | Baiyun district | 0.0 (0.0-0.0) | –1.24 (–2.30 to –0.19) | .02 | –1.36 (–1.92 to –0.80) | <.001 |
|  |  |  | Liwan district | 0.0 (0.0-0.0) | –2.63 (–3.55 to –1.70) | <.001 | –2.55 (–4.67 to –0.42) | .02 |
|  |  |  | Yuexiu district | 0.0 (0.0-0.0) | –1.11 (–2.17 to –0.06) | .04 | –0.94 (–1.26 to –0.61) | <.001 |
|  | **Service delivery** | | |  |  |  |  |  |
|  |  | **Turnaround time for HIV test results, n (%)** | | | | | | |
|  |  |  | Within 1 day | 0.0 (0.0-0.0) | Reference | — | — | — |
|  |  |  | More than 1 day | 0.0 (0.0-0.0) | –0.56 (–1.81 to 0.69) | .39 | — | — |
|  |  | **Availability of VCT services on weekends, n (%)** | | | | | | |
|  |  |  | No | 0.0 (0.0-0.0) | Reference | — | — | — |
|  |  |  | Yes | 0.0 (0.0-0.9) | 0.05 (–0.78 to 0.88) | .90 | — | — |
|  |  | **Number of times counselors unable to provide VCT services for personal reasons last year, n (%)** | | | | | | |
|  |  |  | 0 | 0.0 (0.0-0.0) | Reference | — | — | — |
|  |  |  | <5 | 0.0 (0.0-0.2) | –0.09 (–0.92 to 0.75) | .84 | — | — |
|  |  |  | ≥5 | 0.0 (0.0-0.0) | –0.55 (–2.32 to 1.22) | .54 | — | — |
|  |  | **Posttest gifts for testers, n (%)** | | | | | | |
|  |  |  | No | 0.0 (0.0-0.0) | Reference | — | Reference | — |
|  |  |  | Yes | 0.0 (0.0-0.0) | 2.25 (1.81 to 2.70) | <.001 | 1.20 (–0.52 to 2.91) | .17 |
| **Counselor profiles** | | | | | | | | |
|  | **Age (years), n (%)** | | | | | | | |
|  |  | ≤35 | | 0.0 (0.0-0.0) | Reference | — | — | — |
|  |  | >35 | | 0.0 (0.0-0.0) | –0.29 (–1.10 to 0.52) | .48 | — | — |
|  | **Gender, n (%)** | | | | | | | |
|  |  | Male | | 0.0 (0.0-0.0) | Reference | — | — | — |
|  |  | Female | | 0.0 (0.0-1.0) | 0.03 (–1.04 to 1.10) | .96 | — | — |
|  | **Educational level: bachelor’s degree or higher, n (%)** | | | | | | | |
|  |  | No | | 0.0 (0.0-0.0) | Reference | — | — | — |
|  |  | Yes | | 0.0 (0.0-0.0) | 0.39 (–0.56 to 1.34) | .42 | — | — |
|  | **Major in Public Health, n (%)** | | | | | | | |
|  |  | No | | 0.0 (0.0-0.2) | –0.31 (–1.13 to 0.51) | .46 | — | — |
|  |  | Yes | | 0.0 (0.0-0.0) | Reference | — | — | — |
|  | **Work experience (years), n (%)** | | | | | | | |
|  |  | ≤3 | | 0.0 (0.0-0.3) | Reference | — | — | — |
|  |  | >3 | | 0.0 (0.0-0.0) | 0.03 (–0.80 to 0.86) | .95 | — | — |
|  | **Proportion of VCT services within the counselor's workload, n (%)** | | | | | | | |
|  |  | ≤50% | | 0.0 (0.0-0.0) | Reference | — | Reference | — |
|  |  | >50% | | 0.0 (0.0-0.0) | 1.43 (–0.13 to 2.99) | .08 | 0.62 (–0.11 to 1.35) | .099 |

^a^All models were adjusted by the population under the jurisdiction of each PHC.

^b^β: unadjusted β.

^c^β_m_: multivariate-adjusted β.

^d^VCT: voluntary counseling and testing.

^e^Not applicable.

Table S3. Sensitivity analysis excluding Liwan district: Unadjusted and multivariable analysis^a^ of the facility-level associations of institutional details and counselor profiles with HIV testing volume among primary health care centers (PHCs) in the urban area of Guangzhou, China, 2022.

| Variables | | | | Facility-level HIV testing volume, median (IQR) | IRR^b^ (95%CI) | *P* value | IRR_m_^c^ (95%CI) | *P* value |
| --- | --- | --- | --- | --- | --- | --- | --- | --- |
| **Institutional details** | | | | | | | | |
|  | **Organizational structure** | | | | | | | |
|  |  | **Ownership, n (%)** | |  |  |  |  |  |
|  |  |  | Public ownership | 70 (35-130) | Reference | —^e^ | — | — |
|  |  |  | Private ownership | 75 (65-105) | 0.87 (0.63-1.20) | .40 | — | — |
|  |  | **Institutional prioritization of VCT^d^ services in routine practice, n (%)** | | | | | | |
|  |  |  | No | 68 (35-89) | Reference | — | — | — |
|  |  |  | Yes | 86 (50-129) | 1.23 (0.90-1.66) | .19 | — | — |
|  | **Human resources** | | |  |  |  |  |  |
|  |  | **Number of counselors, n (%)** | | | | | | |
|  |  |  | 1 | 71 (47-108) | Reference | — | — | — |
|  |  |  | 2 or 3 | 75 (50-129) | 1.13 (0.85-1.52) | .41 | — | — |
|  |  | **Counselor turnover in the past year, n (%)** | | | | | | |
|  |  |  | No | 74 (47-114) | Reference | — | — | — |
|  |  |  | Yes | 68 (48-102) | 1.04 (0.73-1.48) | .84 | — | — |
|  | **Infrastructure** | | |  |  |  |  |  |
|  |  | **Dedicated phlebotomy rooms, n (%)** | | | | | | |
|  |  |  | No | 71 (52-111) | Reference | — | — | — |
|  |  |  | Yes | 80 (39-126) | 0.97 (0.72-1.29) | .81 | — | — |
|  |  | **Dedicated and private counseling rooms, n (%)** | | | | | | |
|  |  |  | No | 68 (34-86) | Reference | — | — | — |
|  |  |  | Yes | 87 (50-136) | 1.24 (0.90-1.71) | .20 | — | — |
|  | **Location** | | |  |  |  |  |  |
|  |  | **Walking time to the nearest subway station (minutes), n (%)** | | | | | | |
|  |  |  | ≤15 | 68 (37-108) | Reference | — | — | — |
|  |  |  | >15 | 87 (50-115) | 1.07 (0.78-1.47) | .68 | — | — |
|  |  | **Administrative district, n (%)** | | | | | | |
|  |  |  | Tianhe district | 52 (44-68) | — | — | — | — |
|  |  |  | Haizhu district | 131 (84-253) | — | — | — | — |
|  |  |  | Baiyun district | 111 (102-136) | — | — | — | — |
|  |  |  | Yuexiu district | 40 (22-71) | — | — | — | — |
|  | **Service delivery** | | |  |  |  |  |  |
|  |  | **Turnaround time for HIV test results, n (%)** | | | | | | |
|  |  |  | Within 1 day | 74 (48-114) | Reference | — | — | — |
|  |  |  | More than 1 day | 68 (44-99) | 0.98 (0.68-1.42) | .92 | — | — |
|  |  | **Availability of VCT services on weekends, n (%)** | | | | | | |
|  |  |  | No | 68 (34-104) | Reference | — | — | — |
|  |  |  | Yes | 102 (68-136) | 1.00 (0.70-1.43) | .99 | — | — |
|  |  | **Number of times counselors were unable to provide VCT services for personal reasons last year, n (%)** | | | | | | |
|  |  |  | 0 | 86 (61-129) | Reference | — | Reference | — |
|  |  |  | <5 | 68 (46-113) | 0.84 (0.62-1.14) | .26 | 0.83 (0.64-1.09) | .18 |
|  |  |  | ≥5 | 50 (22-105) | 0.65 (0.42-0.99) | .045 | 0.72 (0.49-1.07) | .11 |
|  |  | **Posttest gifts for testers, n (%)** | | | | | | |
|  |  |  | No | 71 (64-171) | Reference | — | — | — |
|  |  |  | Yes | 73 (46-113) | 0.76 (0.36-1.62) | .48 | — | — |
| **Counselor profiles** | | | | | | | | |
|  | **Age (years), n (%)** | | |  |  |  |  |  |
|  |  | ≤35 | | 74 (34-107) | Reference | — | Reference | — |
|  |  | >35 | | 72 (55-114) | 1.35 (1.01-1.80) | .04 | 1.16 (0.89-1.51) | .27 |
|  | **Gender, n (%)** | | |  |  |  |  |  |
|  |  | Male | | 61 (34-131) | Reference | — | — | — |
|  |  | Female | | 79 (54-110) | 1.03 (0.76-1.41) | .85 | — | — |
|  | **Educational level：Bachelor’s degree or higher, n (%)** | | | | | | | |
|  |  | No | | 75 (48-99) | Reference | — | — | — |
|  |  | Yes | | 72 (46-129) | 1.02 (0.71-1.45) | .93 | — | — |
|  | **Major in Public Health, n (%)** | | | | | | | |
|  |  | No | | 76 (53-113) | 1.01 (0.75-1.37) | .93 | — | — |
|  |  | Yes | | 70 (35-120) | Reference | — | — | — |
|  | **Work experience (years), n (%)** | | | | | | | |
|  |  | ≤3 | | 69 (50-111) | Reference | — | — | — |
|  |  | >3 | | 73 (40-116) | 1.05 (0.78-1.43) | .74 | — | — |
|  | **Proportion of VCT services within the counselor's workload, n (%)** | | | | | | | |
|  |  | ≤50% | | 74 (48-122) | Reference | — | Reference | — |
|  |  | >50% | | 70 (23-100) | 0.64 (0.40-1.02) | .06 | 0.72 (0.49-1.07) | .11 |

^a^All models were adjusted by the population under the jurisdiction of each PHC.

^b^IRR: unadjusted incidence rate ratio, IRR =e^β^.

^c^IRR_m_: multivariate-adjusted incidence rate ratio.

^d^VCT: voluntary counseling and testing.

^e^Not applicable.

Table S4. Sensitivity analysis excluding Liwan district: Unadjusted and multivariable analysis^a^ of the facility-level associations of institutional details and counselor profiles with HIV positivity rate among primary health care centers (PHCs) offering VCT services in the urban area of Guangzhou, China, 2022.

| Variables | | | | Facility-level HIV positivity rate, median (IQR), % | β^b^ (95%CI) | *P* value | β_m_^c^ (95%CI) | *P* value |
| --- | --- | --- | --- | --- | --- | --- | --- | --- |
| **Institutional details** | | | | | | | | |
|  | **Organizational structure** | | |  |  |  |  |  |
|  |  | **Ownership, n (%)** | | | | | | |
|  |  |  | Public ownership | 0.0 (0.0-0.0) | Reference | —^e^ | — | — |
|  |  |  | Private ownership | 0.0 (0.0-1.9) | 0.25 (–0.56 to 1.06) | .54 | — | — |
|  |  | **Institutional prioritization of VCT^d^ services in routine practice, n (%)** | | | | | | |
|  |  |  | No | 0.0 (0.0-0.0) | Reference | — | — | — |
|  |  |  | Yes | 0.0 (0.0-0.9) | –0.79 (–1.80 to 0.21) | .12 | — | — |
|  | **Human resources** | | |  |  |  |  |  |
|  |  | **Number of counselors, n (%)** | | | | | | |
|  |  |  | 1 | 0.0 (0.0-0.0) | Reference | — | — | — |
|  |  |  | 2 or 3 | 0.0 (0.0-0.0) | 0.12 (–0.70 to 0.94) | .77 | — | — |
|  |  | **Counselor turnover in the past year, n (%)** | | | | | | |
|  |  |  | No | 0.0 (0.0-0.0) | Reference | — | — | — |
|  |  |  | Yes | 0.0 (0.0-0.0) | –0.34 (–1.41 to 0.72) | .53 | — | — |
|  | **Infrastructure** | | |  |  |  |  |  |
|  |  | **Dedicated phlebotomy rooms, n (%)** | | | | | | |
|  |  |  | No | 0.0 (0.0-0.0) | Reference | — | Reference | — |
|  |  |  | Yes | 0.0 (0.0-0.0) | –0.76 (–1.51 to –0.01) | .048 | 0.85 (0.60 to 1.11) | <.001 |
|  |  | **Dedicated and private counseling rooms, n (%)** | | | | | | |
|  |  |  | No | 0.0 (0.0-0.0) | Reference | — | Reference | — |
|  |  |  | Yes | 0.0 (0.0-0.0) | –1.33 (–1.98 to –0.68) | <.001 | –1.47 (–1.74 to –1.20) | <.001 |
|  | **Location** | | |  |  |  |  |  |
|  |  | **Walking time to the nearest subway station (minutes), n (%)** | | | | | | |
|  |  |  | ≤15 | 0.0 (0.0-0.0) | Reference | — | — | — |
|  |  |  | >15 | 0.0 (0.0-0.0) | 0.07 (–0.75 to 0.89) | .86 | — | — |
|  |  | **Administrative district, n (%)** | | | | | | |
|  |  |  | Tianhe district | 0.0 (0.0-0.0) | Reference | — | Reference | — |
|  |  |  | Haizhu district | 0.0 (0.0-1.3) | –0.21 (–1.08 to 0.67) | .64 | –0.53 (–0.80 to –0.27) | <.001 |
|  |  |  | Baiyun district | 0.0 (0.0-0.0) | –1.24 (–2.30 to –0.19) | .02 | –1.36 (–1.95 to –0.77) | <.001 |
|  |  |  | Yuexiu district | 0.0 (0.0-0.0) | –1.11 (–2.17 to –0.06) | .04 | –0.94 (–1.25 to –0.63) | <.001 |
|  | **Service delivery** | | |  |  |  |  |  |
|  |  | **Turnaround time for HIV test results, n (%)** | | | | | | |
|  |  |  | Within 1 day | 0.0 (0.0-0.0) | Reference | — | — | — |
|  |  |  | More than 1 day | 0.0 (0.0-0.0) | –0.56 (–1.81 to 0.69) | .38 | — | — |
|  |  | **Availability of VCT services on weekends, n (%)** | | | | | | |
|  |  |  | No | 0.0 (0.0-0.0) | Reference | — | — | — |
|  |  |  | Yes | 0.0 (0.0-0.9) | 0.05 (–0.78 to 0.88) | .90 | — | — |
|  |  | **Number of times counselors unable to provide VCT services for personal reasons last year, n (%)** | | | | | | |
|  |  |  | 0 | 0.0 (0.0-0.0) | Reference | — | — | — |
|  |  |  | <5 | 0.0 (0.0-0.4) | –0.09 (–0.92 to 0.75) | .84 | — | — |
|  |  |  | ≥5 | 0.0 (0.0-0.0) | –0.55 (–2.32 to 1.22) | .54 | — | — |
|  |  | **Posttest gifts for testers, n (%)** | | | | | | |
|  |  |  | No | 0.0 (0.0-0.0) | Reference | — | Reference | — |
|  |  |  | Yes | 0.0 (0.0-0.0) | 2.23 (1.70 to 2.76) | <.001 | 1.17 (–0.25 to 2.59) | .11 |
| **Counselor profiles** | | | | | | | | |
|  | **Age (years), n (%)** | | | | | | | |
|  |  | ≤35 | | 0.0 (0.0-0.0) | Reference | — | — | — |
|  |  | >35 | | 0.0 (0.0-0.0) | –0.29 (–1.10 to 0.52) | .48 | — | — |
|  | **Gender, n (%)** | | | | | | | |
|  |  | Male | | 0.0 (0.0-0.0) | Reference | — | — | — |
|  |  | Female | | 0.0 (0.0-1.0) | 0.03 (–1.04 to 1.10) | .96 | — | — |
|  | **Educational level: bachelor’s degree or higher, n (%)** | | | | | | | |
|  |  | No | | 0.0 (0.0-0.0) | Reference | — | — | — |
|  |  | Yes | | 0.0 (0.0-0.0) | 0.39 (–0.56 to 1.34) | .42 | — | — |
|  | **Major in Public Health, n (%)** | | | | | | | |
|  |  | No | | 0.0 (0.0-0.2) | –0.31 (–1.13 to 0.51) | .46 | — | — |
|  |  | Yes | | 0.0 (0.0-0.0) | Reference | — | — | — |
|  | **Work experience (years), n (%)** | | | | | | | |
|  |  | ≤3 | | 0.0 (0.0-0.5) | Reference | — | — | — |
|  |  | >3 | | 0.0 (0.0-0.0) | 0.03 (–0.80 to 0.86) | .95 | — | — |
|  | **Proportion of VCT services within the counselor's workload, n (%)** | | | | | | | |
|  |  | ≤50% | | 0.0 (0.0-0.0) | Reference | — | Reference | — |
|  |  | >50% | | 0.0 (0.0-0.0) | 1.43 (–0.13 to 2.99) | .07 | 0.62 (–0.10 to 1.35) | .097 |

^a^All models were adjusted by the population under the jurisdiction of each PHC.

^b^β: unadjusted β.

^c^β_m_: multivariate-adjusted β.

^d^VCT: voluntary counseling and testing.

^e^Not applicable.
